# Detecting Tuberculosis-Consistent Findings in Lateral Chest X-Rays Using an Ensemble of CNNs and Vision Transformers

**DOI:** 10.3389/fgene.2022.864724

**Published:** 2022-02-24

**Authors:** Sivaramakrishnan Rajaraman, Ghada Zamzmi, Les R. Folio, Sameer Antani

**Affiliations:** ^1^ Computational Health Research Branch, National Library of Medicine, National Institutes of Health, Bethesda, MD, United States; ^2^ Moffitt Cancer Center, Tampa, FL, United States

**Keywords:** chest radiographs, CNN, deep learning, tuberculosis classification and localization, vision transformers, ensemble learning, significance analysis

## Abstract

Research on detecting Tuberculosis (TB) findings on chest radiographs (or Chest X-rays: CXR) using convolutional neural networks (CNNs) has demonstrated superior performance due to the emergence of publicly available, large-scale datasets with expert annotations and availability of scalable computational resources. However, these studies use only the frontal CXR projections, i.e., the posterior-anterior (PA), and the anterior-posterior (AP) views for analysis and decision-making. Lateral CXRs which are heretofore not studied help detect clinically suspected pulmonary TB, particularly in children. Further, Vision Transformers (ViTs) with built-in self-attention mechanisms have recently emerged as a viable alternative to the traditional CNNs. Although ViTs demonstrated notable performance in several medical image analysis tasks, potential limitations exist in terms of performance and computational efficiency, between the CNN and ViT models, necessitating a comprehensive analysis to select appropriate models for the problem under study. This study aims to detect TB-consistent findings in lateral CXRs by constructing an ensemble of the CNN and ViT models. Several models are trained on lateral CXR data extracted from two large public collections to transfer modality-specific knowledge and fine-tune them for detecting findings consistent with TB. We observed that the weighted averaging ensemble of the predictions of CNN and ViT models using the optimal weights computed with the Sequential Least-Squares Quadratic Programming method delivered significantly superior performance (MCC: 0.8136, 95% confidence intervals (CI): 0.7394, 0.8878, *p* < 0.05) compared to the individual models and other ensembles. We also interpreted the decisions of CNN and ViT models using class-selective relevance maps and attention maps, respectively, and combined them to highlight the discriminative image regions contributing to the final output. We observed that (i) the model accuracy is not related to disease region of interest (ROI) localization and (ii) the bitwise-AND of the heatmaps of the top-2-performing models delivered significantly superior ROI localization performance in terms of mean average precision [mAP@(0.1 0.6) = 0.1820, 95% CI: 0.0771,0.2869, *p* < 0.05], compared to other individual models and ensembles. The code is available at https://github.com/sivaramakrishnan-rajaraman/Ensemble-of-CNN-and-ViT-for-TB-detection-in-lateral-CXR.

## 1 Introduction

Artificial intelligence (AI) methods, particularly deep learning (DL)-based convolutional neural network (CNN) models, have demonstrated remarkable performance in natural and medical computer vision applications (Schmidhuber, 2015). Considering chest-X-ray (CXR) analysis, CNN models have outperformed conventional machine learning (ML) methods for semantic segmentation, classification, and object detection, among other tasks (Wang et al., 2017; Irvin et al., 2019; Bustos et al., 2020).

Research on detecting Tuberculosis (TB)-consistent findings in CXRs using DL methods has demonstrated superior performance due to the emergence of publicly available, large-scale datasets with expert annotations and availability of scalable computational resources (Jaeger et al., 2014; Lakhani and Sundaram, 2017; Sivaramakrishnan et al., 2018; Pasa et al., 2019; Rajaraman and Antani, 2020). However, these studies only use the frontal CXR projections, i.e., the posterior-anterior (PA), and the anterior-posterior (AP) views, for analysis and decision-making. To the best of our knowledge, lateral CXR projections have, heretofore, not been used for AI detection approaches to pulmonary diseases before this work. Lateral CXR projections of children with clinically suspected pulmonary TB, in addition to the conventional frontal projections, are critical and showed an increase in the detection sensitivity of enlarged lymph nodes by 1.8% and specificity by 2.5% (Swingler et al., 2005). Further, the World Health Organization (WHO) recommends the use of lateral CXR projections to identify mediastinal or hilar lymphadenopathy (World Health Organization, 2016), especially in younger children with primary TB where a bacteriological confirmation might be challenging. As discussed in (Gaber et al., 2005), lateral CXRs provide useful spatial diagnostic information on the thoracic cage, pleura, lungs, pericardium, heart, mediastinum, and upper abdomen and help identify lymphadenopathy in children with primary TB (Gaber et al., 2005). Another study (Herrera Diaz et al., 2020) discusses the current national Canadian guidelines suggesting using lateral CXR projections for TB screening upon admission to long-term care facilities. These studies underscore the importance of using lateral CXR projections as they carry useful information on disease manifestation and progression; hence, this study aims to explore these least studied types of CXR projection (the lateral) and propose a novel approach for detecting TB-consistent findings.

Recently, Vision Transformers (ViTs) (Zhai et al., 2021) with built-in self-attention mechanisms have demonstrated comparable performance to CNNs in natural and medical visual recognition tasks, while requiring fewer computational resources. Several studies (Liu and Yin, 2021; Shome et al., 2021; Park et al., 2022) used ViTs to improve pulmonary disease detection in frontal CXRs to detect manifestations consistent with COVID-19 disease. Another study (Duong et al., 2021) used a ViT model to detect TB-consistent findings in frontal CXRs and obtained an accuracy of 97.72%. The promising performance of ViT models in medical visual recognition tasks is constrained by sparse data availability (Zhai et al., 2021). Unlike CNN models, ViT models lack intrinsic biases, i.e., the properties of translation equivariance, which is the similarity in processing different image parts regardless of their absolute position, and they do not consider the relationship between the neighboring image pixels. Further, the computational complexity of ViT models increases with the input image resolution resulting in demand for a higher resource. In contrast, CNN models have shown promising performance even with limited data due to their inherent inductive bias characteristics that help in convergence and generalization. However, CNN models do not encode the relative position of different image features and may require large receptive fields to encode the combination of these features and capture long-range dependencies in an input image. This leads to increased convolutional kernel sizes and subsequently the computational complexity (Alzubaidi et al., 2021). A potential solution could be to exploit the advantages of both models, i.e., CNNs and ViTs toward decision-making for the task under study.

Several ensemble methods including majority voting, averaging, weighted averaging, and stacking, have been studied for medical visual recognition tasks (Dietterich, 2000). Considering CXR analysis, particularly TB detection, ensemble methods have been widely used to improve performance in semantic segmentation, classification, and object detection tasks (Hogeweg et al., 2010; Ding et al., 2017; Islam et al., 2017; Rajaraman et al., 2018a; Rajaraman and Antani, 2020). However, to the best of our knowledge, we are not aware of studies that perform an ensemble of ViTs or an ensemble of both CNN and ViT models for disease detection, particularly detecting TB-consistent findings using lateral CXRs. The main contribution of this work is a systematic approach that benefits from constructing ensembles of the best models from both worlds (i.e., CNNs and ViTs) to detect TB-consistent findings using lateral CXRs through reduced prediction variance and improved performance.

The steps in this systematic study can be summarized as follows: (i) First, ImageNet-pretrained CNN models, viz, VGG-16 (Simonyan and Zisserman, 2015), DenseNet-121 (Huang et al., 2017), and EfficientNet-V2-B0 (Tan and Le, 2021) and the ImageNet-pretrained ViT models, viz, ViT-B/16, ViT-B/32, ViT-L/16, and ViT-L/32 (Zhai et al., 2021) are retrained on a combined selection of publicly available lateral CXR collections (Rajpurkar et al., 2017; Bustos et al., 2020). This step is performed to convert the weight layers specific to the lateral CXR modality and learn to classify normal and abnormal lateral CXRs; (ii) Next, the retrained models are used to transfer the lateral CXR modality-specific knowledge to improve performance in the related task of classifying lateral CXRs as showing no abnormalities or other findings that are consistent with TB; (iii)The predictions of the top-K (K = 2, 3, 5, 7) models are combined using several ensemble methods such as majority voting, simple averaging, and weighted averaging using the optimal weights derived with the Sequential Least-Squares Quadratic Programming (SLSQP) algorithm (Gupta and Gupta, 2018). We construct a “model-level” ensemble of the CNN and ViT models by flattening, concatenating the features from their deepest layers, and adding the classification layers to classify the lateral CXRs to their respective categories; (iv) We also interpret CNN and ViT model decisions through the use of class-selective relevance maps (CRM) (Kim et al., 2019) and attention maps, respectively, and construct an ensemble of these heatmaps and attention maps using several ensemble methods. Finally, we analyze and report statistical significance in the results obtained using the individual models and their ensembles using confidence intervals (CIs) and *p* values.

## 2 Materials and Methods

### 2.1 Datasets

The following publicly available datasets are used in this study:

CheXpert CXR dataset: The authors in (Irvin et al., 2019) released a collection of frontal and lateral CXR projections, showing normal lungs, and other pulmonary abnormalities. The dataset contains 224,316 CXRs collected from 65,240 patients at the Stanford University Hospital in California. The CXRs are labeled using a natural language processing (NLP)-based automatic labeler for the presence of 14 thoracic abnormalities mentioned in radiological reports. The collection includes 23,633 lateral CXRs manifesting various pulmonary abnormalities and 4,717 lateral CXRs showing no abnormalities. In this study, the lateral CXR projections are split at the patient level into 90/10 proportions for the train and test sets and are used during CXR modality-specific pretraining.

PadChest CXR dataset: A collection of 160,000 frontal and lateral CXRs and their associated radiological reports are released by (Bustos et al., 2020). The collection includes normal and abnormal CXRs collected from 67,000 patients at the San Juan Hospital in Spain. The CXR images are automatically labeled for 174 radiographic findings, based on the Unified Medical Language System (UMLS) terminology. The collection includes 33,454 lateral CXRs manifesting several pulmonary abnormalities and 14,229 lateral CXRs showing no abnormalities. The abnormal lateral CXR collection also includes 530 CXRs collected from patients diagnosed with TB. The set of CXRs manifesting TB-consistent findings and an equal number of lateral CXRs with no abnormalities are used during the fine-tuning. The ground truth annotations for the hold-out test set consisting of 53 images, and showing findings that are consistent with TB, are provided by an expert radiologist (with >30 years of experience). The radiologist used the web-based VGG Image Annotator tool (VIA, Oxford, England) (Dutta and Zisserman, 2019) to annotate the test collection by manually setting boundary boxes for what is believed to be TB-consistent findings. Table 1 shows the datasets, the numbers of images, and their respective patient-level train/test splits used in this study. The lateral CXR images from the PadChest and CheXpert collections are resized to 224 × 224 pixel dimensions to reduce computational overhead.

**TABLE 1:** Datasets and their respective patient-level train/test splits. Data in parenthesis denotes the 90/10 train/test splits. A part of the lateral CXRs in the PadChest CXR collection that show no abnormalities and those with TB-consistent manifestations are used for fine-tuning. The rest of the data from the PadChest and CheXpert lateral CXR collections are used for CXR modality-specific pretraining.

Dataset	CXR modality-specific pretraining		Fine-tuning	
	Abnormal	Normal	TB	Normal
PadChest	32923 (29631/3292)	13698 (12328/1370)	530 (477/53)	530 (477/53)
CheXpert	23633 (21270/2363)	4717 (4245/472)	-	-

### 2.2 Classification Models

The following CNN and ViT Models are used in this study: (i) VGG-16 (Simonyan and Zisserman, 2015); (ii) DenseNet-121 (Huang et al., 2017); (iii) EfficientNet-V2-B0 (Tan and Le, 2021); (iv) ViT-Base (B)/16 (Zhai et al., 2021); (v) ViT-B/32 (Zhai et al., 2021); (vi) ViT-Large (L)/16 (Zhai et al., 2021); and (vii) ViT-L/32 (Zhai et al., 2021). The CNN models are selected based on their superior performance in CXR-based visual recognition tasks (Wang et al., 2017; Rajaraman et al., 2018b; Irvin et al., 2019; Rajaraman et al., 2020a). The numbers 16 and 32 in the ViT models denote the size of input image patches. The length of the input image patch sequence is inversely proportional to the square of the patch size. Thus, the ViT models with smaller patch sizes are computationally more expensive (Zhai et al., 2021). Interested readers are referred to (Wang et al., 2017; Rajaraman et al., 2018b; Irvin et al., 2019; Rajaraman et al., 2020a; Zhai et al., 2021) for a detailed description of these models' architecture.

### 2.3 CXR Modality-Specific Pretraining, Fine-Tuning, and Ensemble Learning

**FIGURE 1:**
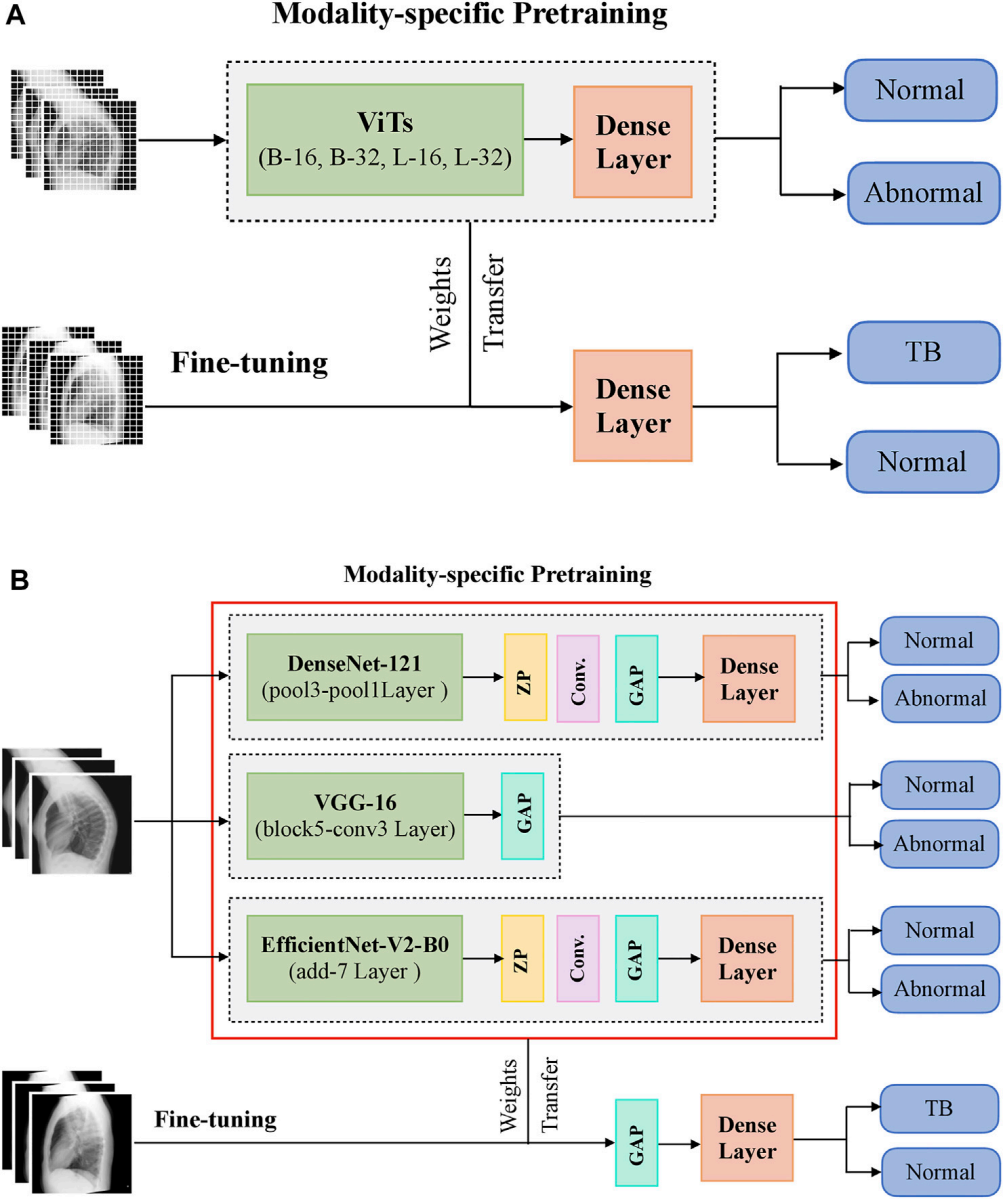
A systematic approach of training the models during CXR modality-specific pretraining and fine-tuning stages. **(A)** ViTs and **(B)** CNNs.

During CXR modality-specific pretraining, the CNN models are instantiated with their ImageNet pretrained weights, truncated at their optimal intermediate layers (Rajaraman et al., 2020b), and appended with the following layers: (i) a zero-padding (ZP) layer, (ii) a convolutional layer with 512 filters, each of size 3 × 3, (iii) a global averaging pooling (GAP) layer; and (iv) a final dense layer with two nodes and Softmax activation. The optimal intermediate layers are identified from pilot analyses for the task under study. The ViT models are instantiated with their pretrained weights learned from a combined selection of ImageNet and Imagenet21K datasets. These models are then truncated at the output classification token layer and appended with a flattening layer and a final dense layer with two nodes to output prediction probabilities. Figure 1 shows the block diagram of models used in CXR modality-specific pretraining and fine-tuning stages.

The CNN and ViT models are then retrained on a combined selection of lateral CXRs from the CheXpert and PadChest datasets (Table 1). This process is called CXR modality-specific pretraining and it is performed to impart CXR modality-specific knowledge to (i) coarsely learn the characteristics of normal and abnormal lateral CXRs and (ii) convert the weight layers learned from natural images to the input CXR modality. The modality-specific pretrained CNN and ViT models are then fine-tuned to classify the lateral CXRs as showing no abnormalities or other findings that are consistent with TB. The datasets are split at the patient level into 90% for training and 10% for testing during the CXR modality-specific pretraining and finetuning stages as shown in Table 1. We allocated 10% of the training data for validation with a fixed seed. The training data is augmented using affine transformations such as rotation (−5, +5), horizontal flipping, width, and height shifting (−5, +5), and normalized so the image pixel values lie in the range (0, 1). During CXR modality-specific pretraining, the CNN and ViT models are trained for 100 epochs, using a stochastic gradient descent (SGD) optimizer with an initial learning rate of 1e-2 and momentum of 0.9, to minimize the categorical cross-entropy loss. We used callbacks to store model checkpoints and reduced the learning rate whenever the validation loss ceased to decrease. The best-performing model, delivering the least validation loss at the end of the training epochs is stored to predict the hold-out test set. During fine-tuning, the CXR modality-specific pretrained models are finetuned using the SGD optimizer with an initial learning rate of 1e-4 and momentum of 0.9. We used callbacks for early stopping and learning rate reduction. The best-performing model, delivering the least validation loss at the end of the training epochs is stored to predict the hold-out test set.

**FIGURE 2:**
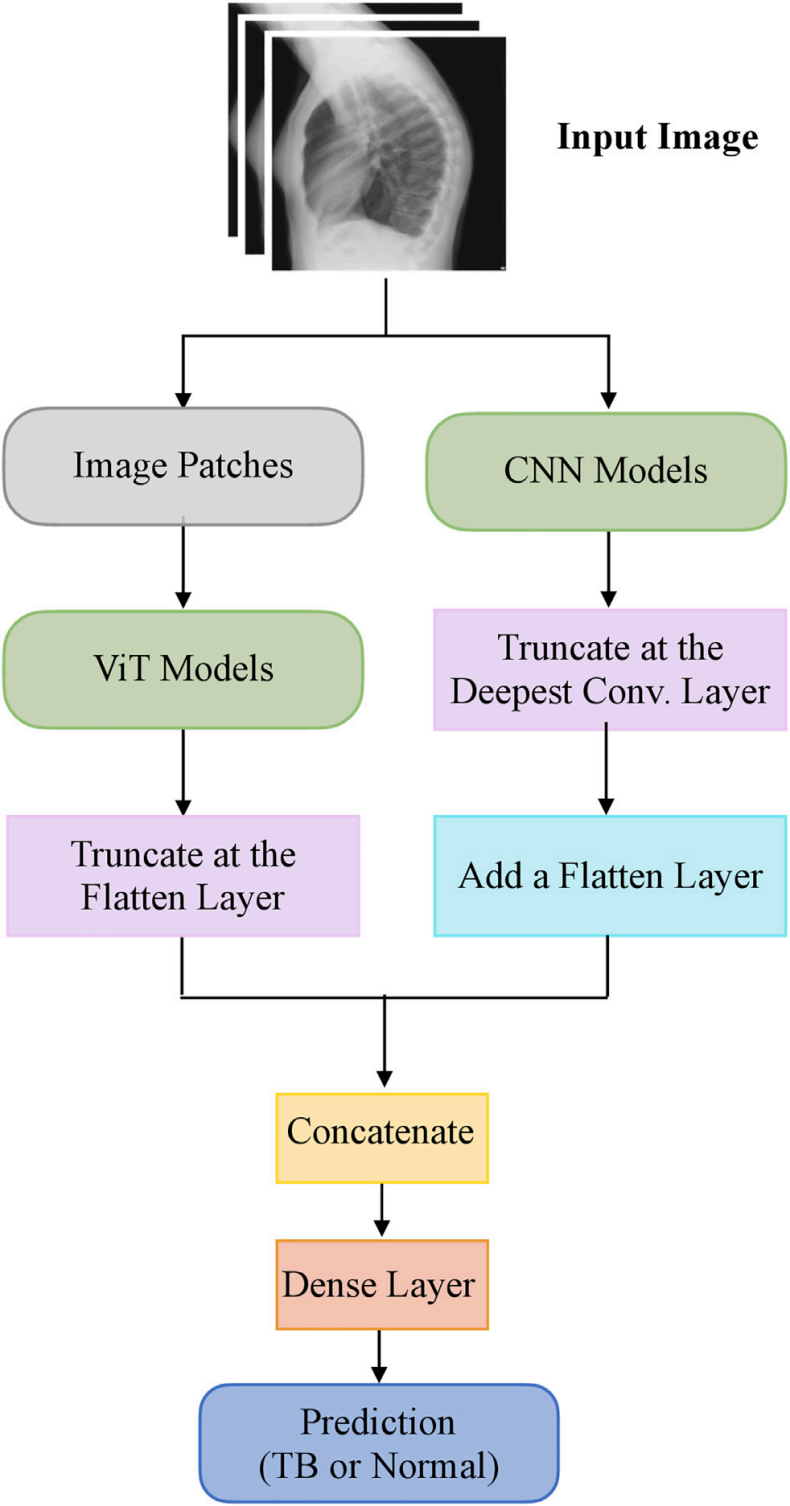
A model-level ensemble constructed using fine-tuned CNN and ViT models.

The top-K (K = 2, 3, 5, 7) fine-tuned models that deliver superior performance with the hold-out test set are used to construct ensembles. We constructed “prediction-level” and “model-level” ensembles. At the prediction level, we used several ensemble strategies such as majority voting, simple averaging, and SLSQP-based weighted averaging to combine the top-K model predictions. For SLSQP-based weighted averaging, we computed the optimal weights by minimizing the total logarithmic loss using the SLSQP algorithm (Gupta and Gupta, 2018) to help convergence. For the model-level ensemble, the top-K models are instantiated with their fine-tuned weights. The ViT models are truncated at the flatten layer. The CNN models are truncated at their deepest convolutional layer and added with a flatten layer. The output from the flattened layers of the ViT and CNN models are then concatenated and appended with the final dense layer to output class probabilities. The weights of trainable layers are frozen and only the final dense layer is trained to output probabilities of classifying the lateral CXRs into normal or TB categories. The model-level ensemble is trained using an SGD optimizer and an initial learning rate of 1e-5. Callbacks are used to store model checkpoints and reduce the learning rate whenever the validation performance did not improve. The best-performing model with the least validation loss is stored to predict the hold-out test set. Figure 2 illustrates the construction of model-level ensembles using the fine-tuned CNN and ViT models. The performance of the models during CXR modality-specific pretraining, fine-tuning, and ensemble learning are evaluated using the following metrics: (i) accuracy; (ii) area under the receiver-operating-characteristic curve (AUROC); (iii) area under the precision-recall curve (AUPRC); (iv) precision; (v) recall; (vi) F-score; (vii) Matthews correlation coefficient (MCC), (viii) Diagnostic Odds Ratio (DOR), and (ix) Cohen’s Kappa. These metrics are expressed in Eqs 1–11.

$$Accuracy=\frac{TP+TN}{TP+TN+FP+FN}$$ (1)

$$Recall=\frac{TP}{TP+FN}$$ (2)

$$Precision=\frac{TP}{TP+FP}$$ (3)

$$F-score=2\times \frac{Precision\times Recall}{Precision+Recall}$$ (4)

$$MCC=\frac{TP\times TN-FP\times FN}{{\left(\left(TP+FP\right)\left(TP+FN\right)\left(TN+FP\right)\left(TN+FN\right)\right)}^{1/2}}$$ (5)

$$DOR=\frac{\left(TP\times TN\right)}{\left(FP\times FN\right)}$$ (6)

$$P_{o}=\frac{\left(TP+TN\right)}{\left(TP+FP+FN+TN\right)}$$ (7)

$$P_{true}=\frac{\left(TP+FN\right)\left(TP+FP\right)}{{\left(TP+FP+FN+TN\right)}^{2}}$$ (8)

$$P_{false}=\frac{\left(FP+TN\right)\left(FN+TN\right)}{{\left(TP+FP+FN+TN\right)}^{2}}$$ (9)

$$P_{e}=P_{true}+P_{false}$$ (10)

$$Cohen^{'}s\;Kappa=\frac{\left(P_{o}-P_{e}\right)}{1-P_{e}}$$ (11)

Here, TP, TN, FP, and FN denote the true positive, true negative, false positive, and false negative values, respectively. The models are trained and evaluated using Tensorflow Keras version 2.6.2 on a Linux system with NVIDIA GeForce GTX 1080 Ti GPU, and CUDA dependencies for GPU acceleration.

### 2.4 Model Explainability

DL models are often criticized for their “black box” behavior, i.e., lack of explanations toward their predictions. This lack of explainability could be attributed to (i) their architectural depth that may not allow decomposability into explainable components and (ii) the presence of non-linear layers that perform complex data transformations and result in non-deterministic behavior that adversely impacts clinical interpretations. Methods have been proposed (Selvaraju et al., 2017) to explain model predictions by highlighting discriminative parts of the image that causes the model to classify the images to their respective categories. In this study, we used class-selective relevance maps (CRM) (Kim et al., 2019) to discriminate image regions used by the fine-tuned CNN models to categorize the CXRs as showing TB-consistent findings. It has been reported that the CRM-based visualization (Kim et al., 2019) outperformed the conventional gradient-based class activation maps (Selvaraju et al., 2017) in interpreting model predictions.

We computed the attention maps from the fine-tuned ViT models using the attention rollout method discussed in (Zhai et al., 2021). The steps involved in computing the attention map consists of (i) getting the attention weights from each transformer block, (ii) averaging the attention weights across all the heads, (iii) adding an identity matrix to the attention matrix to account for residual connections, (iv) re-normalizing the weights and recursively multiplying the weight matrices to mix the attention across tokens through all the layers, and (v) computing the attention from the output token to the input space. The bounding box coordinates of the heatmaps and attention maps are computed as follows: (i) A difference binary image is generated using the original input lateral CXR image and the heatmap/attention map-overlaid image; (ii) the polygonal coordinates of the connected components in the binary image are measured that gives the coordinates of the vertices and that of the line segments making up the sides of the polygon, and (iii) a binary mask is generated from the polygon and the coordinates are stored for further analysis. The delineated ROIs are compared against the ground truth annotations provided by the radiologist.

For evaluating localization performance, we used several ensemble methods, such as simple averaging, SLSQP-based weighted averaging, and a bitwise-AND of the heatmaps and attention maps of top-K performing models. In simple averaging, the heatmaps and attention maps obtained respectively using the CNN and ViT models are averaged to produce the final heatmap, highlighting discriminative ROIs toward TB detection. In SLSQP-based weighted averaging, the optimal weights obtained using the SLSQP method are used while averaging the heatmaps and attention maps. In a bitwise-AND ensemble, the heatmaps and attention maps are binarized and bitwise-ANDed. The corresponding pixel in the final heatmap is activated only if there is complete agreement among activations in the candidate heatmaps and attention maps. The ROI localization performance of the constituent models and their ensembles is measured in terms of the mean average precision (mAP) metric. The mAP is calculated by taking the mean precision over 11 IoU threshold values within the range [0.1, 0.6] at equal intervals of 0.05 [denoted as mAP@[0.1, 0.6]] (GTUA et al., 2014).

### 2.5 Statistical Significance Analysis

It has been reported in (Diong et al., 2018) that 90–96% of the studies published in scientific journals do not measure statistical significance in the reported results, casting doubt on algorithm reliability and confidence. In this study, we analyzed statistical significance using the 95% confidence intervals (CIs) for the MCC metric measured as the Clopper–Pearson binomial CI interval. For RoI localization, we measured the 95% CIs measured as the Clopper–Pearson binomial CI interval for the mAP metric achieved by the individual models and their ensembles to report statistical significance. The StatsModels and SciPy Python packages are used in this analysis. We obtained the *p-*value from the CIs using the methods reported in (Altman and Bland, 2011). Considering the upper and lower limits of the 95% CI as *u* and *l* respectively, the standard error (SE) is measured as given in Eq. 12.

SE=(u−l)(2×1.96) (12)

The test statistic *z* is given by Eq. 13

$$z=\frac{Diff}{SE}$$ (13)

Here, *Diff* denotes the estimated differences between the models for the measured metric.

The *p*-value is then calculated as given in Eq 14.

$$p=exp\left(-0.717\times z-0.416\times z^{2}\right)$$ (14)

## 3 Results

### 3.1 CXR Modality-Specific Pretraining and Fine-Tuning

Recall that the CNN and ViT models are instantiated with their ImageNet-pretrained weights and retrained on a combined selection of lateral CXRs from the CheXpert and PadChest datasets. The test performance achieved during CXR modality-specific pretraining is shown in Table 2. From Table 2, we observed the following: (i) The training time for CNN models is comparatively small than ViT models. The EfficientNet-V2-B0 model took the least while the ViT-L/16 model took the most time for training and convergence. (ii) The VGG-16 model demonstrated superior performance in terms of accuracy, F-score, MCC, DOR, Kappa, AUROC, and AUPRC metrics. The EfficientNet-V2-B0 model demonstrated superior recall and ViT-B/32 demonstrated superior precision compared to other models. However, considering a balanced measure of precision and recall, as provided by the MCC metric, the VGG-16 model demonstrated superior performance compared to other models. (iii) We observed that the 95% CIs obtained for the MCC metric using the VGG-16 model are not significantly different (*p* > 0.05) from other models. Due to this lack of statistical significance, all modality-specific pretrained models are fine-tuned to evaluate performance in the TB classification task. Table 3 shows the performance achieved by the fine-tuned models that classify the lateral CXRs as showing no abnormalities or other abnormalities that are consistent with TB.

**TABLE 2:** Test performance achieved by the CNN and ViT models during lateral CXR modality-specific pretraining. The values in parenthesis denote the 95% CI measured as the Clopper–Pearson binomial interval for the MCC metric. Bold numerical values denote superior performance.

Model	Accuracy	Recall	Precision	F	MCC	DOR	Kappa	AUROC	AUPRC	Training time (seconds)
ViT-B/16	0.7747	0.7988	0.8913	0.8425	0.4596 (0.3647,0.5545)	9	0.4512	0.8276	0.9334	17582.14
ViT-B/32	0.7394	0.7151	**0.9218**	0.8054	0.4621 (0.3671,0.5571)	**11**	0.4293	0.8375	0.9375	10739.29
ViT-L/16	0.7678	0.7846	0.8946	0.8360	0.4555 (0.3606,0.5504)	9	0.4442	0.8276	0.9332	54949.73
ViT-L/32	0.7872	0.8324	0.8792	0.8552	0.4584 (0.3635,0.5533)	9	0.4560	0.8364	0.9373	28797.83
EfficientNet-V2-B0	0.7794	**0.8391**	0.8645	0.8516	0.4231 (0.3290,0.5172)	8	0.4223	0.8152	0.9281	2296.54
VGG-16	**0.8009**	0.8361	0.8931	**0.8637**	**0.4998 (0.4046,0.5950)**	**11**	**0.4960**	**0.8526**	**0.9441**	9316.52
DenseNet-121	0.7886	0.8230	0.8885	0.8545	0.4747 (0.3796,0.5698)	10	0.4701	0.8401	0.9393	7281.22

**TABLE 3:** Performance achieved by the fine-tuned models toward the TB classification task. The values in parenthesis denote the 95% CI measured as the Clopper–Pearson binomial interval for the MCC metric. Bold numerical values denote superior performance.

Model	Accuracy	Recall	Precision	F	MCC	DOR	Kappa	AUROC	AUPRC	Training time (seconds)
ViT-B/16	0.7642	0.6792	0.8182	0.7422	0.5361 (0.4411,0.6311)	12	0.5283	0.8548	0.8668	828.30
ViT-B/32	0.8302	0.7547	0.8889	0.8163	0.6680 (0.5783,0.7577)	30	0.6604	0.9227	0.9351	338.46
ViT-L/16	0.8302	**0.8302**	0.8302	0.8302	0.6604 (0.5702,0.7506)	24	0.6604	0.8943	0.9084	1539.06
ViT-L/32	0.7736	0.7170	0.8085	0.7600	0.5507 (0.4560,0.6454)	12	0.5472	0.8786	0.8911	574.24
EfficientNet-V2-B0	0.8019	0.6981	0.8810	0.77900	0.6172 (0.5246,0.7098)	22	0.6038	0.8896	0.9025	114.89
VGG-16	0.8208	0.7358	0.8864	0.8041	0.6510 (0.5602,0.7418)	27	0.6415	0.9110	0.9219	267.40
DenseNet-121	**0.8585**	0.8113	**0.8958**	**0.8515**	**0.7202 (0.6347,0.8057)**	**41**	**0.7170**	**0.9288**	**0.9423**	313.44

**FIGURE 3:**
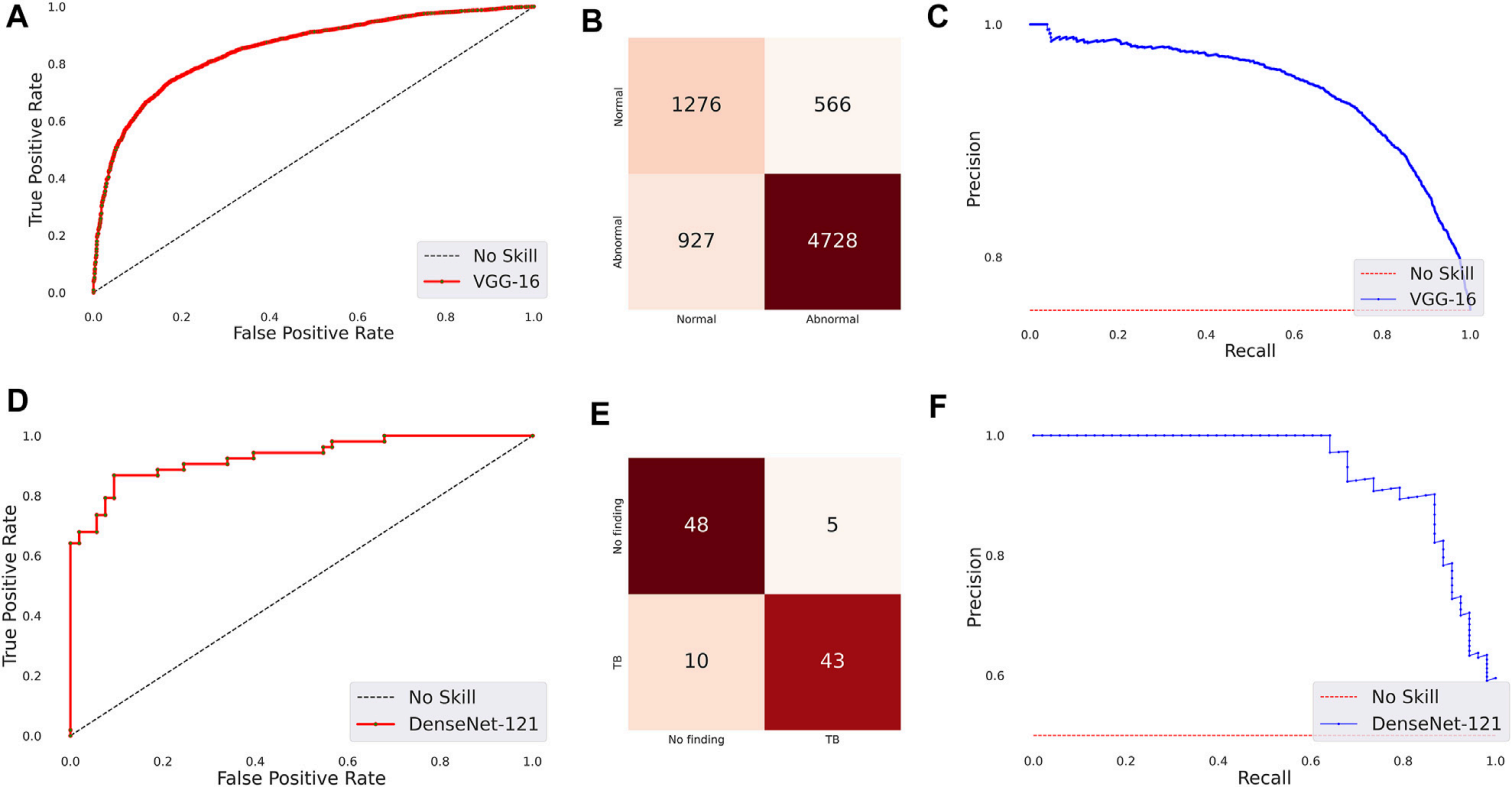
Performance curves achieved by the models used in this study. CXR modality-specific pretraining (VGG-16): **(A)** AUROC; **(B)** AUPRC; **(C)** Confusion matrix. Fine-tuning (DenseNet-121): **(D)** AUROC; **(E)** AUPRC, and **(F)** Confusion matrix.

The following are observed from Table 3: (i) The CNN models took comparatively lesser time to converge than the ViT models. This observation is analogous to CXR modality-specific pretraining. (ii) The DenseNet-121 model demonstrated superior performance in terms of accuracy, precision, F-score, MCC, DOR, Kappa, AUROC, and AUPRC metrics. The ViT-L/16 model demonstrated superior recall compared to other models. However, considering the MCC metric, the DenseNet-121 model demonstrated superior performance compared to other models. (iii) The 95% CIs for the MCC metric achieved by the DenseNet-121 model demonstrated a tighter error margin, hence higher precision, compared to other models. We observed that the MCC metric achieved by the DenseNet-121 model is significantly superior to ViT-B/16 (*p* = 0.0001), ViT-L/32 (*p* = 0.0002), and EfficientNet-V2-B0 (*p* = 0.0183) models. We also observed that the MCC metric achieved by the VGG-16 model is significantly superior to the ViT-B/16 (*p* = 0.0133) and ViT-L/32 (*p* = 0.0304) models. These observations underscore the fact that the CNN models delivered superior classification performance compared to the ViT models. Figure 3 shows the AUROC, AUPRC, and confusion matrices achieved by the VGG-16 and DenseNet-121 models during the CXR modality-specific pretraining and fine-tuning stages, respectively. A no-skill classifier fails to discriminate between the classes and would predict a random or a constant class in all circumstances.

**FIGURE 4:**
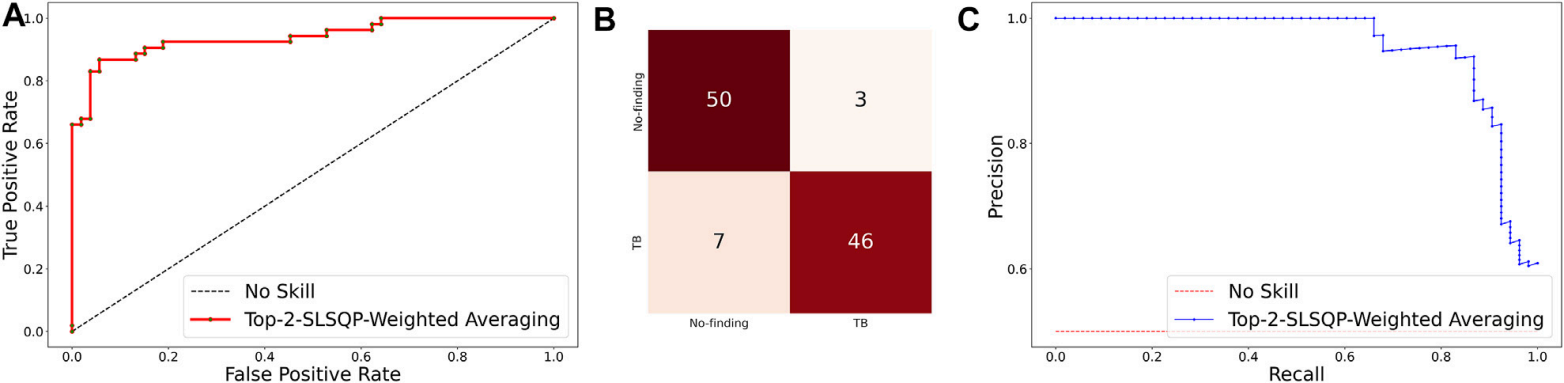
Performance curves achieved using SLSQP-based weighted averaging of the predictions of top-2 fine-tuned models, i.e., DenseNet-121, and ViT-B/32 models. **(A)** AUROC; **(B)** Confusion matrix, and **(C)** AUPRC.

The ensemble of the top-K models (K = 2, 3, 5, 7) is constructed to evaluate any improvement in classification performance during fine-tuning. Table 4 shows the performance achieved using various ensemble methods discussed in this study. From Table 4, we observe that the performance obtained through SLSQP-based weighted averaging is comparatively higher than other ensembles and their constituent models. This demonstrates that, unlike using equal weights, the use of optimal weights to combine the predictions of constituent models improved classification performance. (ii) The SLSQP-based weighted averaging [optimal weights: (0.65, 0.35)] of the predictions of the top-2 fine-tuned models, viz. DenseNet-121 and ViT-B/32 delivered superior performance in terms of accuracy, Kappa, and significantly superior performance in terms of the MCC metric (0.8136, 95% CI: (0.7394, 0.8878)) compared to its constituent models, viz. DenseNet-121 (*p* = 0.0137), and ViT-B/32 (*p* = 0.0002). This ensemble also demonstrated significantly superior performance in terms of MCC metric compared to other models, viz. VGG-16 (*p* = 0.0001), EfficientNet-V2-B0 (*p* = 0.0001), ViT-B/16 (*p* = 0.0001), ViT-L/16 (*p* = 0.0001), and ViT-L/32 (*p* = 0.0001) models. The model-level ensemble of the top-2 fine-tuned models, i.e., DenseNet-121 and ViT-B/32 demonstrated superior values for the DOR metric. Figure 4 shows the AUROC, AUPRC, and confusion matrices achieved by the SLSQP-based weighted averaging of the predictions of the top-2 fine-tuned models.

**TABLE 4:** Test performance obtained using prediction-level and model-level ensembles. The values in parenthesis denote 95% CI for the MCC metric measured as the Clopper-Pearson binomial interval. Bold numerical values denote superior performance.

Ensemble	Models	Accuracy	Recall	Precision	F-score	MCC	DOR	Kappa	AUROC	AUPRC	Training time (seconds)
Majority voting	Top-2	0.8774	**0.8868**	0.8704	0.8785	0.7549 (0.6730,0.8368)	51	0.7547	0.8774	0.9069	NA
	Top-3	0.8679	0.8302	0.898	0.8628	0.738 (0.6542,0.8218)	47	0.7358	0.8679	0.9065	NA
	Top-5	0.8585	0.7925	0.913	0.8485	0.7233 (0.6381,0.8085)	47	0.717	0.8585	0.9046	NA
	Top-7	0.8585	0.7925	0.913	0.8485	0.7233 (0.6381,0.8085)	47	0.717	0.8585	0.9046	NA
Simple averaging	Top-2	0.8679	0.8113	0.9149	0.86	0.7406 (0.6571,0.8241)	53	0.7358	0.9388	0.9525	NA
	Top-3	0.8491	0.8113	0.8776	0.8431	0.7001 (0.6128,0.7874)	34	0.6981	0.9377	0.9515	NA
	Top-5	0.8679	0.8113	0.9149	0.86	0.7406 (0.6571,0.8241)	53	0.7358	0.937	0.949	NA
	Top-7	0.8396	0.7925	0.875	0.8317	0.6823 (0.5936,0.7710)	30	0.6792	0.9313	0.9441	NA
SLSQP-weighted averaging	Top-2	**0.9057**	0.8679	0.9388	0.902	**0.8136 (0.7394,0.8878)**	110	**0.8113**	0.9409	0.9542	NA
	Top-3	**0.9057**	**0.8868**	0.9216	**0.9039**	0.8119 (0.7375,0.8863)	96	**0.8113**	0.9352	0.9492	NA
	Top-5	0.8962	0.8491	0.9375	0.8911	0.796 (0.7192,0.8728)	94	0.7925	0.9388	0.952	NA
	Top-7	**0.9057**	0.8679	0.9388	0.902	**0.8136 (0.7394,0.8878)**	110	**0.8113**	0.937	0.9503	NA
Model-level	Top-2	0.8962	0.8113	0.9773	0.8866	0.8041 (0.7285,0.8797)	**223**	0.7925	**0.9491**	**0.9587**	91.4263
	Top-3	0.8679	0.7736	**0.9535**	0.8542	0.7493 (0.6667,0.8319)	87	0.7358	0.9274	0.9433	418.05
	Top-5	0.8679	0.7736	**0.9535**	0.8542	0.7493 (0.6667,0.8319)	87	0.7358	0.9427	0.9525	555.088
	Top-7	0.8585	0.7547	0.9524	0.8421	0.7329 (0.6486,0.8172)	79	0.717	0.9366	0.9493	758.957

### 3.2 Evaluating TB-Consistent ROI Localization Performance

**FIGURE 5:**
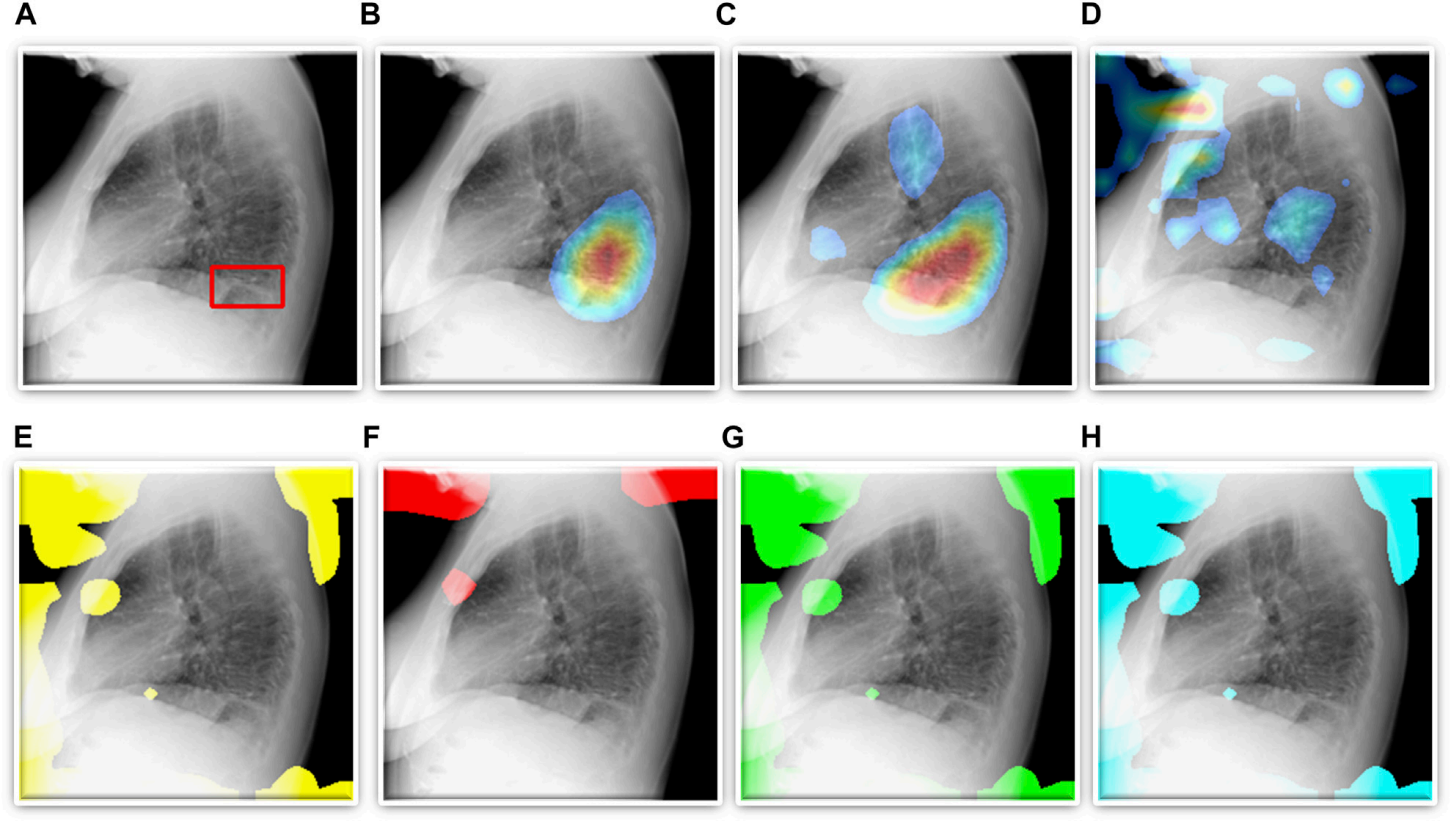
TB-consistent ROI localization achieved using the fine-tuned models. **(A)** An instance of lateral CXR with expert-annotated ROI consistent with TB (shown with a red bounding box); **(B)** VGG-16; **(C)** DenseNet-121; **(D)** EfficientNet-V2-B0; **(E)** ViT-B/16; **(F)** ViT-B/32; **(G)** ViT-L/16, and **(H)** ViT-L/32.

As described in Section 2.4, we use CRMs and attention maps to interpret the predictions of the CNN and ViT models, respectively. The delineated ROIs are compared against the ground truth annotations provided by the radiologist. Figure 5 shows a sample lateral CXR with expert-annotated ROI consistent with TB and the discriminative ROIs highlighted by the fine-tuned CNN and ViT models discussed in this study. Table 5 shows the TB-consistent ROI localization performance in terms of mAP metric, achieved by the individual models.

**TABLE 5:** TB-consistent ROI localization performance achieved by the fine-tuned CNN and ViT models. The values in parenthesis denote the 95% CI measured as the Clopper-Pearson binomial interval for the mAP metric. Bold numerical values denote superior performance.

Model	mAP@[0.1, 0.6]
ViT-B/16	0.0573 (0,0.1205)
ViT-B/32	0.0567 (0,0.1196)
ViT-L/16	0.0573 (0,0.1205)
ViT-L/32	0.0573 (0,0.1205)
EfficientNet-V2-B0	0.0690 (0.0001,0.1379)
VGG-16	**0.1283 (0.0374,0.2192)**
DenseNet-121	0.1052 (0.0218,0.1886)

**FIGURE 6:**
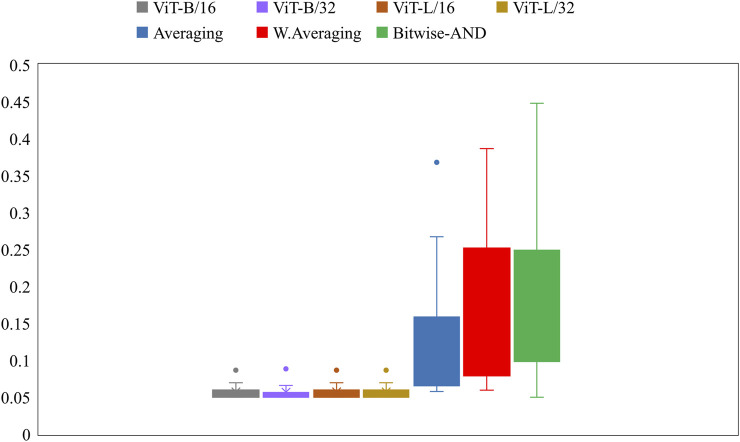
Box plots showing the range of mAP values obtained by the individual models and other ensembles.

Further, we constructed ensembles of the heatmaps of the top-2 models from Table 5, viz. VGG-16 and DenseNet-121 models using simple averaging, SLSQP-based weighted averaging, and bitwise-AND techniques. Figure 6 shows the box plots for the range of mAP values achieved by the individual models and other ensembles. Table 6 shows the TB-consistent ROI localization performance achieved in terms of the mAP metric by the model ensembles.

**TABLE 6:** TB-consistent ROI localization performance achieved by the model ensembles. The values in parenthesis denote the 95% CI measured as the exact Clopper-Pearson binomial interval for the mAP metric. Bold numerical values denote superior performance.

Model	mAP@[0.1, 0.6]
Simple averaging	0.1332 (0.0408,0.2256)
SLSQP-weighted averaging	0.1742 (0.0711,0.2773)
Bitwise-AND	**0.1820 (0.0771,0.2869)**

**FIGURE 7:**
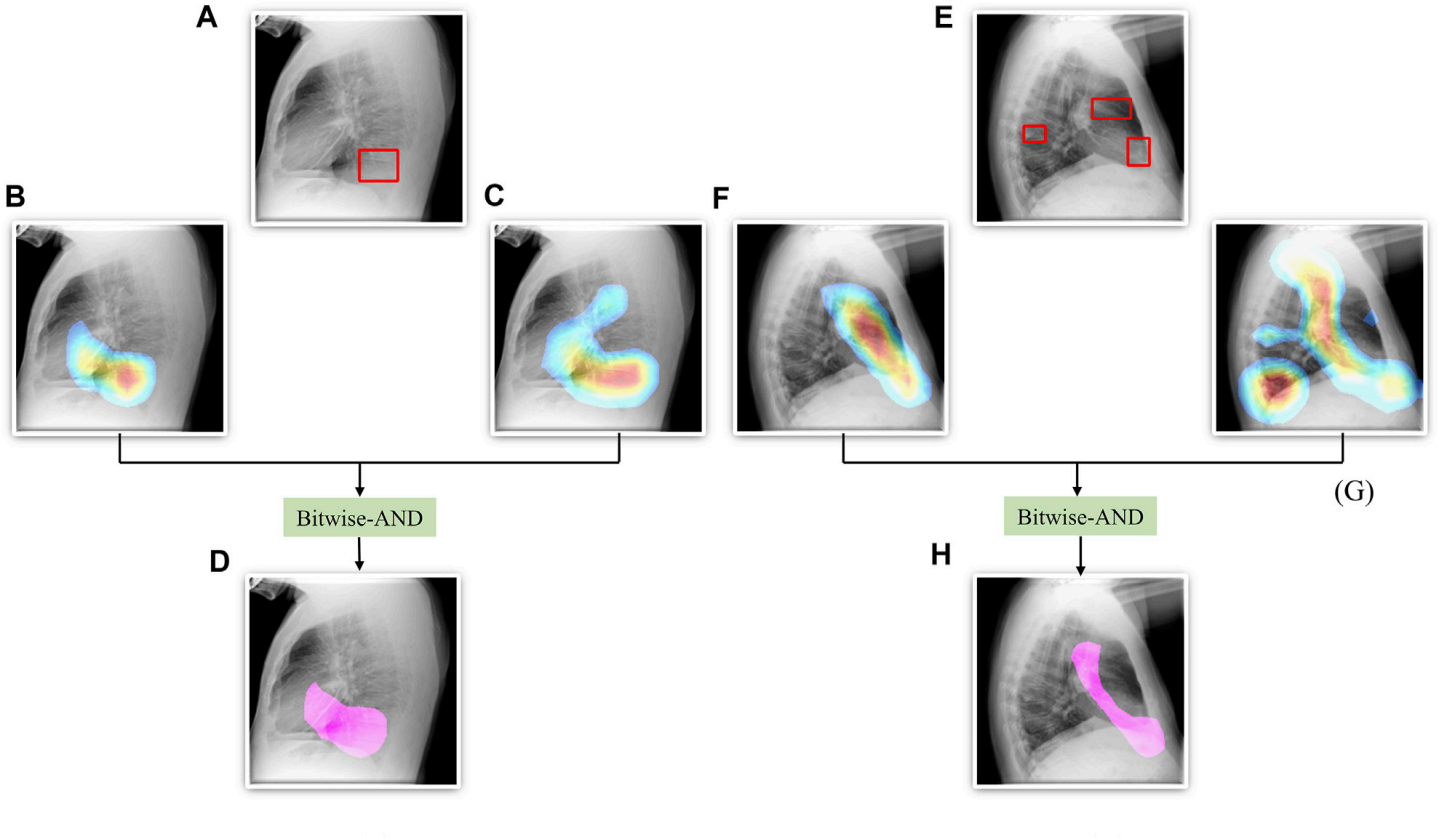
A Bitwise-AND ensemble generated using the heatmaps produced by the top-2 performing models, viz. VGG-16 and DenseNet-121 models. **(A)** and **(E)** Sample test lateral CXRs with expert ground truth annotations (shown in red bounding box); **(B)** and **(F)** Heatmaps produced by the VGG-16 model; **(C)** and **(G)** Heatmaps produced by the DenseNet-121 model, and **(D)** and **(H)** Mask resulting from the Bitwise-AND operation of the heatmaps produced by the VGG-16 and DenseNet-121 models.

From Figure 6, we observe that the maximum, mean, median, the total range, and the inter-quartile range of the mAP values achieved with the Bitwise-AND ensemble is significantly higher (*p* < 0.05) than those obtained with the ViT models and considerably higher than the averaging and weighted averaging ensembles. From Table 6, we observe that all ensemble methods demonstrated superior values for the mAP metric compared to the individual models (Table 5). The bitwise-AND operation resulted in superior values for the mAP metric compared to the constituent models, other models, and ensembles. The mAP metric achieved by the bitwise-AND ensemble is observed to be significantly superior to ViT-B/16, ViT-L/16, ViT-L/32 (*p* = 0.0199), ViT-B/32 (*p* = 0.0193), and EfficientNet-V2-B0 (*p* = 0.0014) models. This performance is followed by the SLSQP-based weighted averaging ensemble that demonstrated significantly superior localization performance compared to ViT-B/16, ViT-L/16, ViT-L/32 (*p* = 0.0264), and EfficientNet-V2-B0 (*p* = 0.0029) models. Figure 7 shows a Bitwise-AND ensemble of the heatmaps produced by the top-2 models, viz. VGG-16 and DenseNet-121 models, for instances of test images.

## 4 Discussion

Following findings from our pilot studies which are consistent with prior observations [34], the ImageNet-pretrained CNNs with their total depth and the ImageNet-pretrained ViT models demonstrated sub-optimal performance toward the task of TB detection. Therefore, we truncated the ImageNet-pretrained CNN models at their optimal intermediate layers, appended them with the classification layers. Further, instead of using ImageNet weights learned from stock photographic images we trained the CNN and ViT models on a large-scale collection of lateral CXR data. These CXR modality-specific pretrained weights serve as a promising initialization to promote modality-specific knowledge transfer and improved adaptation and performance of the models in the relevant task of detecting TB-consistent manifestations.

From our findings and evaluation results, we observe that the ViT models demonstrate sub-optimal classification and ROI localization performance and significantly higher training time, compared to the CNN-based DL models. These findings confirm our suspicion that these may be due to the lack of intrinsic inductive biases. On the other hand, CNN models show superior performance at lower training times even with our limited dataset. Even though CheXpert and PadChest data sets have a cumulative of over 384,316 CXRs only 76,033 lateral CXRs are found in them with only 530 lateral CXRs (0.13% of the total number of lateral CXRs) exhibiting manifestations consistent with TB. This could be a significant factor in the sub-optimal performance exhibited by the ViT models. We improved both classification and ROI localization performance, qualitatively and quantitatively, using CXR modality-specific training, fine-tuning, and constructing model ensembles. This performance improvement with ensemble learning is consistent with the literature (He et al., 2016; Rajaraman et al., 2018a; Rajaraman et al., 2019).

We also show that classification performance is not indicative of reliable disease prediction. For example, even though the average classification performance of ViT models is approximately 80%, their average MAP score is only 5.7% which is evident from the visualization studies, examples of which are shown in Figures 5E–H. This underscores the need for visualization of localized disease prediction regions to verify model credibility.

Regarding the use of ensembles, we find in the literature a frequent use of methods such as majority voting, simple averaging, and weighted averaging with equal eights. However, we show that using optimized weighting using specialized techniques, such as SLSQP, result in significantly superior classification performance, e.g., the SLSQP accuracy achieved with the top-2 models is 0.9057 compared to 0.8679 for simple averaging (*p* = 0.0001). Similar behavior is observed for localization performance as well.

Our study has the following limitations: (i) Lateral CXRs help confirm abnormal opacification spatial location, however, have more overlapping structures (e.g., shoulders including scapula and humeral heads), decreasing conspicuity relative to frontal projections. Given that there are more frontal projection CXRs available with TB manifestations, we provide an avenue to explore the combination including lateral images that we believe will improve performance. (ii) There are a very small number of lateral CXRs with TB-consistent findings available for fine-tuning the models which have, very likely, affected the sub-par performance of ViT models as they demand more training data and training time due to their functional characteristics. We expect that the performance of the models would scale with increased data and appropriate empowerment of computational resources. (iii) There is also an imbalance in the number of left or right lateral CXRs in an already small dataset of 530 TB disease-positive images. On the positive side, through augmentation, ensemble learning, and optimized weighting of model predictions, we were able to achieve a lateral-view agnostic performance that was significantly high. However, it is important to consider that the anatomical view presented in a left lateral image is different from the one presented in the other. For clinical diagnostic or screening applications, it would be necessary to train the classifier on these differences so that a reliable and robust interpretation of the prediction can be obtained. Further, research is ongoing in building combination model architectures like ConViT (d’Ascoli et al., 2021) that combines characteristics of the CNN and ViT architectures toward improving performance. Such models should be studied in future studies.

## Data Availability

The original contributions presented in the study are included in the article/supplementary material, further inquiries can be directed to the corresponding author.
